# Lipoxin A4 Inhibits NLRP3 Inflammasome Activation in Rats With Non-compressive Disc Herniation Through the JNK1/Beclin-1/PI3KC3 Pathway

**DOI:** 10.3389/fnins.2020.00799

**Published:** 2020-09-17

**Authors:** Jin Jin, Yonggang Xie, Cunxian Shi, Jiahai Ma, Yihao Wang, Leyan Qiao, Kezhong Li, Tao Sun

**Affiliations:** ^1^Department of Pain Management, Shandong Provincial Hospital, Cheeloo College of Medicine, Shandong University, Jinan, China; ^2^Department of Anesthesiology, The Affiliated Yantai Yuhuangding Hospital of Qingdao University, Yantai, China; ^3^Department of Anesthesiology, Qingdao Municipal Hospital, Qingdao, China

**Keywords:** lipoxin A4, non-compressive disc herniation, NLRP3, JNK1, beclin-1, PI3KC3

## Abstract

**Background:**

Non-compressive disc herniation is induced by an inflammatory response from the nucleus pulposus tissue and nerve roots. Lipoxins (LXs) are important endogenous anti-inflammatory mediators in the body, helping to inhibit neutrophil recruitment and stimulate autophagy in monocytes and macrophages. Here, we investigated the molecular mechanisms underlying the effects of exogenous lipoxin administration on rats with non-compressive disc herniation.

**Method:**

A non-compressive disc herniation model was established in rats. Fifty rats were randomly divided into: sham group, model group, PI3K inhibitor (LY294002) group, lipoxin A4 group (LXA4), and PI3K inhibitor and lipoxin A4 group (LY294002 + LXA4). Similar groupings were established for rat spinal neurons. Changes in the mechanical pain threshold and thermal pain threshold were monitored at different times. The expression of proinflammatory and anti-inflammatory mediators was assessed by ELISA, while immunohistochemistry was employed to measure the expression levels of NLRP3 and p-JNK1. The expression levels of autophagy-related proteins were measured by western blot.

**Results:**

*In vivo*, the pain threshold was markedly decreased in the model group at each time point examined compared with that in sham group. LY294002 treatment further reduced the pain threshold. After LXA4 injection, the pain threshold was significantly increased, and the effect of LY294002 was significantly weakened (*p* < 0.05). The levels of proinflammatory cytokines were increased in rats with non-compressive disc herniation, and these levels were further increased by LY294002 treatment (*p* < 0.05). However, treatment with LXA4 significantly reduced the levels of these proinflammatory cytokines in the model group (*p* < 0.05). The opposite effect was observed for anti-inflammatory mediators. The expression of NLRP3 was largely increased in the model group compared with that in the sham group (*p* < 0.05). Treatment with LY294002 also increased the NLRP3 expression level, while the administration of LXA4 elicited the opposite effect. Furthermore, western blot analysis showed that the expression of autophagy-related proteins was greatly decreased in the model group, whereas it was significantly increased in the LXA4 group (*p* < 0.05). The *in vitro* results were consistent with the outcomes observed *in vivo*.

**Conclusions:**

These data suggested that LXA4 inhibited NLRP3 activation in rats with non-compressive disc herniation by regulating the JNK1/beclin-1/PI3KC3 pathway.

## Introduction

Root nerve pain, a painful and protracted condition, greatly affects the quality of life in patients. Disc herniation is the major cause of root nerve pain, and is induced by either mechanical compression or an inflammatory response originating from the nucleus pulposus and nerve roots (non-compressive disc herniation) (Li et al., 2015; Choi et al., 2016). Studies have shown that non-compressive disc herniation plays an important role in root neuralgia, and has attracted significant research attention in recent years (Miao et al., 2015; Liu et al., 2016).

The nucleus pulposus, the cause of non-compressive disk herniation, can be regarded as an “isolated antigen.” It can stimulate immune responses and cause inflammatory reactions once the “antigen” is exposed to the immune system (Olmarker et al., 1995). Neutrophils, mononuclear macrophages, and other inflammatory cells will be recruited and proliferate. The released enzymes and inflammatory cytokines, such as interleukin-1β (IL-1β), IL-6, tumor necrosis factor (TNF), and prostaglandins, will lead to swelling of the nerve roots, vacuolar degeneration, finally resulting in root pain (Zhang and An, 2007). The NLRP3 (nucleotide-binding oligomerization domain-like receptor family pyrin domain-containing 3) inflammasome is recruited following pathogenic or endogenous signals mediated by pattern-recognition receptors (Martinez et al., 2018). Caspase-1 is the main component of the NLRP3 inflammasome, and stimulates the expression of IL-1β and IL-18 (Doyle et al., 2019). When caspase-1 is activated, pro-IL-1β and pro-IL-18 are cleaved, thereby promoting the maturation and secretion of IL-1β and IL-18.

Lipoxins (LXs) are important endogenous anti-inflammatory mediators (Chandrasekharan and Sharma-Walia, 2015) synthesized from arachidonic acid through the activity of lipoxygenases (English et al., 2017). LXs are important “brake signals” for relieving and inhibiting neutrophil recruitment, and stimulate autophagy in monocytes and macrophages (Hughes et al., 2017). They can also regulate cytokine secretion and expression, which greatly contributes to the pathophysiology of chronic pain (Brennan et al., 2018).

For many diseases associated with chronic pain, LXs exert significant regulatory effects on a variety of inflammatory cell types and factors. The beneficial effects of LXs reported to date include the promotion of inflammation regression in acute lung injury (Fang et al., 2015), asthma (Lu et al., 2018), and renal fibrosis (Roach et al., 2015). LXA4, one of the most widely investigated lipoxins, has been reported to effectively inhibit inflammation-related and neuropathic pain (Kim et al., 2011; Martini et al., 2016), with one study showing that intrathecal injection of LXA4 could alleviate neuropathic pain in a rat model of non-compressive lumbar disc herniation (Miao et al., 2015).

However, relatively few studies have investigated the role of LXA4 in non-compressive disc herniation-induced root neuralgia. Consequently, the aim of this study was to investigate the molecular mechanisms underlying the effects of exogenous LXA4 administration on rats with non-compressive disc herniation.

## Materials and Methods

### Experimental Animals

Fifty specific-pathogen-free (SPF)-grade, healthy, female Sprague–Dawley (SD) rats, 8–10 weeks old, weighing 240–320 g, were purchased from Beijing Wei Tong Lihua Experimental Animal Technology Co., Ltd (Beijing, China; license number: SCXK [Beijing] 20160006). The rats were maintained in a controlled environment at a temperature of 23 ± 2°C, humidity of 55 ± 5%, 12-h light–dark cycle, and with *ad libitum* access to food and water. All animal experiments were carried out following NIH guidelines (NIH Pub. No. 85–23, revised 1996), and were reviewed and approved by the Animal Protection and Use Committee of Shandong University.

### Establishment of a Rat Model of Non-compressive Disc Herniation

Rats were first anesthetized by intraperitoneal injection of 10% chloral hydrate (300 mg/kg). Under a sterile environment, a 25–30-mm medial longitudinal incision was made at the midpoint between the two iliac crests, then subcutaneous tissue and paravertebral muscles were incised through blunt separation from the right L4–L5 spinal. It exposes L4/5 facet joints, removes right L4 lamina and articular processes, and exposes L5 nerve roots and dorsal root ganglion (Kim et al., 2011; Miao et al., 2015). The nucleus pulposus (0.5 mg) of the rat’s autologous caudal vertebrae was removed and placed gently at the proximal end of the L5 dorsal root ganglia, and placed in contact with the nerve roots.

### Drug Injection Through an Intrathecal Catheter

Following anesthesia by intraperitoneal injection of 10% chloral hydrate (300 mg/kg), a 2–3 cm incision was made in the middle of the back of the rats to expose the L4–5 intervertebral foramen. A PE-10 catheter was inserted into the L6 spinous process through the exposed intervertebral foramen, and the cerebrospinal fluid flowed out along the catheter, which indicated that implantation was successful. The catheter was fixed and the wound stitched. After recovery from general anesthesia, 3 μL of 2% lidocaine was injected through the catheter, and reversible side limb paralysis after injection was indicative of successful catheter placement.

### Animal Grouping and Medication

**FIGURE 1:**
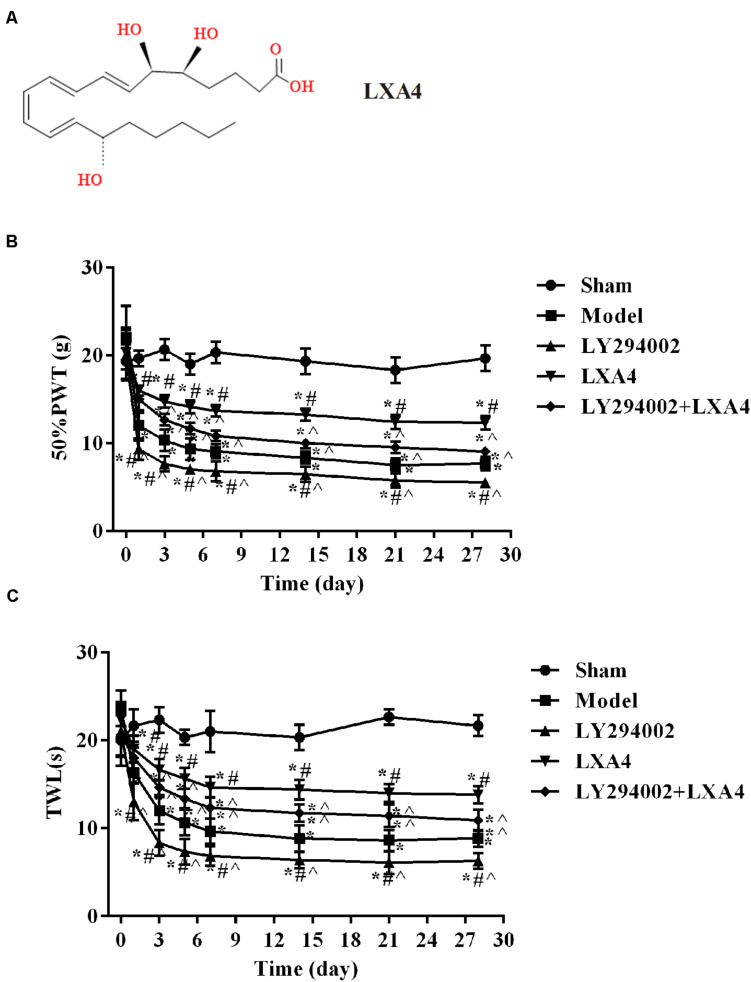
Comparison of 50% mechanical pain threshold (paw withdrawal threshold, PWT) and thermal pain threshold (thermal withdrawal latency, TWL) at different time points in each treatment group. **(A)** The complete stereochemistry of LXA4; **(B)** 50% PWT; **(C)** TWL. *p < 0.05 compared with the sham group; ^#^*p* < 0.05 compared with the model group; ^∧^*p* < 0.05 compared with the LXA4 group. Experiments were repeated three times for each group. Data are presented as means ± standard deviation (SD). One-way analysis of variance (ANOVA) was used for comparisons among groups followed by Dunnett’s *t*-test.

Fifty rats were randomly divided into 5 groups, with 10 rats per group: (i) Sham operation group (sham), which included a sham operation and the injection of 10 μL of saline solution; (ii) model group, which included modeling surgery and the injection of 10 μL of saline solution; (iii) lipoxin group (LXA4) (Miao et al., 2015), which consisted of model group rats injected with LXA4 (10 μL, 100 ng); (iv) PI3K inhibitor (LY294002) group, which comprised modeling surgery and injection of LY294002 (10 μL, 25 mmol/L); and (v) PI3K inhibitor and LXA4 combination group (LY294002 + LXA4), comprising model surgery, followed by injection of LY294002 and then that of LXA4. Each group of rats was injected with the corresponding drugs once a day for 28 consecutive days. LY294002 was purchased from Sigma–Aldrich (St. Louis, MO, United States). LXA4 was purchased from Cayman Chemical Co., United States. The complete stereochemistry of LXA4 is shown in Figure 1A.

### Determination of the Mechanical and Thermal Pain Thresholds

Changes in the mechanical pain threshold (paw withdrawal threshold, PWT) and thermal pain threshold (thermal withdrawal latency, TWL) before and after drug injection were detected before surgery and 1, 3, 5, 7, 14, 21, and 28 days after surgery. In each group, the “up–down” method was used to stimulate the plantar side of the rat’s paw using von Frey hairs with different bending forces. A PWT of 50% was assumed if the rat showed a rapid withdrawal and a cringe in the hind limbs, which was also called a positive reaction. To determine the TWL, the rats were first placed on a 6-mm thick plexiglass plate. The palm of the rat’s hindlimb was irradiated through the plexiglass plate using a thermal pain stimulator. The incubation period from the start of irradiation to hind limb retraction, acting as an indicator of thermal pain, was recorded using an electronic stopwatch. The analgesic effects of the treatments and their duration were also analyzed.

### Specimen Collection

The TWL and 50% PWT were measured on postoperative day 28. The rats were then anesthetized by intraperitoneal injection of 3% sodium pentobarbital (50 mg/kg) and euthanized by cervical dislocation. On ice, an incision was made in the skin of the back of the rats and the L4–L6 spinal dorsal horn was removed. Part of the spinal dorsal horn and dorsal root ganglion was stored in 4% paraformaldehyde, and the remaining tissue was stored in a freezer at -80°C.

### Cell Culture

Rat spinal neurons were purchased from Procell Life Science & Technology Co., Ltd (CP-R144, Wuhai, China) and cultured in DMEM (Gibco, Rockville, MD, United States) supplemented with 10% fetal bovine serum (FBS) (Sigma–Aldrich) and 100 U/mL penicillin/100 mg/mL streptomycin. All cells were incubated at 37°C with 5% CO_2_. Cells were collected and used for experiments in the logarithmic growth phase.

### Cell Model Grouping

Spinal neurons were stimulated with 0.01 μg/mL TNF-α (Chen et al., 2019) and then treated with 100 nM LXA4. These cells were randomly divided into 5 groups: (1) Control (untreated) group; (2) model group: treatment with 0.01 μg/mL TNF-α; (3) LXA4 group: treatment with 100 nM LXA4 and 0.01 μg/mL TNF-α; (4) LY294002 group: treatment with 0.01 μg/mL TNF-α, and then with 20 μM of LY294002 (Xu et al., 2018); and (5) LY294002 + LXA4 group: as for the LY294002 group, plus treatment with 100 nM LXA4. After 48 h, the treated cells were collected for subsequent experiments.

### ELISA

The levels of TNF-α (orb452907), IL-1β (orb453587), IL-18 (orb107403), IL-4 (orb303658), IL-10 (orb76364), and TGF-β1 (orb7087) (all from Biorbyt, Cambridge, United Kingdom) in the spinal dorsal horn and dorsal root ganglion were measured following the instructions of the respective ELISA kits.

### Immunohistochemistry

Tissues (5 μm) were treated with xylene, and hydrated via an ethanol gradient, followed by incubation in 3% H_2_O_2_ at room temperature for 10 min to block endogenous peroxidase activity. The slices were subsequently blocked with 5% goat serum (Gibco) for 20 min, and then incubated with rabbit anti-rat NLRP3 (1:500, DF7438, Affinity Biosciences, Beijing, China) or p-JNK1 (1:500, orb312293, Biorbyt) overnight at 4°C. Samples were then incubated with a horseradish peroxidase-labeled goat anti-rabbit IgG antibody (1:1000, ABIN101988, antibodies-online, Aachen, Germany) at 37°C for 40 min.

Cells grown on glass coverslips were fixed in paraformaldehyde for 30 min and added into 3% H_2_O_2_ for 15–20 min, then followed by 2–3 PBS washes. After blocking (AbDil-Tx; TBS containing 0.1% Triton X-100, 2% BSA, and 0.1% sodium azide) at 37°C for 30 min, cells were incubated with anti-NSE (1:200, ab53025, Abcam, United Kingdom) and anti-p-JNK1 (1:200, orb312293, Biorbyt) antibodies at 37°C for 30 min, washed 2–3 times with PBS, and then incubated with rabbit anti-Goat-IgG (1:800, SA0004-4, Proteintech, Wuhan, China) at 37°C for 30 min.

After washing, the cells were incubated with SABC for 30 min and washed 3 times with PBS, stained with DAB, slightly restained with hematoxylin, dehydrated in an ethanol gradient, made transparent, and then mounted in neutral tree lipid. Finally, the slides were observed under a microscope (IX83, Olympus, Japan) at × 400 magnification.

### Western Blot

Total protein was extracted from both tissues and cells using a total protein extraction kit (cat. no. BC3640-50T; Beijing Solarbio Science & Technology Co., Ltd). Protein concentration was measured using a BCA kit (Solarbio, Beijing, China). A total of 40 μg of each protein sample was mixed with 10% SDS gel buffer at a ratio of 1:1 and the protein denatured by heating at 95°C for 5 min. The PVDF membrane (Merck, Darmstadt, Germany) was rotated at 80 V for 30 min, and then blocked with 5% skimmed milk powder in TBST for 1 h at 4°C. Next, the membranes were incubated overnight at 4°C with rabbit anti-rat NLRP3 (1:500, DF7438, Affinity Biosciences), OX42 (1:500, orb176288), and p-JNK1 (1:500, orb312293); polyclonal rabbit anti-rat MAP1LC3A (1:500, orb378164), MAP1LC3B (1:500, orb382715), beclin1 (1:500, orb227780), PI3KC3 (1:500, orb382585), caspase-1 (1:200, orb213639), and β-actin (1:2000, orb178392) (all from Biorbyt) antibodies diluted in a TBST solution containing 3% BSA. Before testing, the membranes were rewarmed, and then incubated with horseradish peroxidase-labeled goat anti-rabbit IgG (1:1000, ABIN101988, antibodies-online) for 1 h. Then, the membranes were washed with an ECL substrate for 3–5 min. Protein expression levels were normalized to those of β-actin, measured through grayscale scanning, and quantified using ImageJ.

### Statistical Analysis

Data were analyzed using SPSS 19.0, and the results presented as means ± standard deviation (SD). Comparisons between groups were performed by one-way analysis of variance (ANOVA) followed by Dunnett’s *t*-test. A *p*-value < 0.05 was considered to be statistically significant.

## Results

### LXA4 Ameliorated the Pain Threshold in Rats With Non-compressive Disc Herniation

The changes in the PWT (Figure 1B) and TWL (Figure 1C) are shown in Tables 1, 2. Before surgery, there was no significant difference in the 50% PWT and TWL values among all the groups (*p* > 0.05). After surgery, the 50% PWT and TWL were differentially decreased in the spinal surgery groups, with the lowest levels being observed in the LY294002 group (*p* < 0.05). Compared with the sham group, the 50% PWT and TWL were significantly lower in all the surgery groups at each time point evaluated (*p* < 0.05). Notably, the 50% PWT and TWL were significantly higher in the LXA4 group than in the other surgery groups at each time point (*p* < 0.05). These data suggested that LXA4 could improve the pain threshold.

**TABLE 1:** Data for 50% mechanical pain threshold (paw withdrawal threshold, PWT) at different time points in each treatment group.

Time (Day)	Sham group	Model group	LXA4 group	LY294002 group	LY294002+LXA4 group
0	19.33 ± 3.51	22.00 ± 6.25	20.33 ± 1.53	20.00 ± 5.00	22.00 ± 5.00
1	19.67 ± 1.53	12.00 ± 3.00*	16.00 ± 1.00*#	9.33 ± 2.08*#^∧^	15.00 ± 2.64*^∧^
3	20.67 ± 2.08	10.33 ± 2.08*	14.73 ± 1.27*#	7.67 ± 1.53*#^∧^	12.67 ± 1.15*^∧^
5	19.00 ± 2.00	9.33 ± 2.08*	14.13 ± 1.27*#	7.03 ± 0.95*#^∧^	11.67 ± 1.15*^∧^
7	20.33 ± 2.08	9.07 ± 1.41*	13.70 ± 1.13*#	6.80 ± 1.93*#^∧^	10.83 ± 1.05*^∧^
14	19.33 ± 2.52	8.33 ± 1.15*	13.27 ± 1.16*#	6.47 ± 1.50*#^∧^	10.00 ± 1.00*^∧^
21	18.33 ± 2.52	7.60 ± 1.44*	12.43 ± 1.40*#	5.77 ± 1.36*#^∧^	9.53 ± 1.10*^∧^
28	19.67 ± 2.52	7.70 ± 1.37*	12.33 ± 1.40*#	5.50 ± 0.50*#^∧^	9.07 ± 0.81*^∧^

Compared with sham group, *p < 0.05; compared with model group, ^#^*p* < 0.05; compared with LXA4 group, ^∧^*p* < 0.05.

**TABLE 2:** Data for thermal pain threshold (thermal withdrawal latency, TWL) at different time points in each treatment group.

Time (Day)	Sham group	Model group	LXA4 group	LY294002 group	LY294002+LXA4 group
0	21.00 ± 3.61	23.67 ± 3.52	22.33 ± 3.51	21.00 ± 5.00	20.67 ± 4.04
1	21.67 ± 3.21	16.33 ± 2.08*	19.00 ± 2.65*#	13.00 ± 3.60*#^∧^	18.00 ± 2.00*^∧^
3	22.33 ± 2.52	12.00 ± 2.65*	16.67 ± 2.08*#	8.33 ± 2.52*#^∧^	14.67 ± 1.53*^∧^
5	20.33 ± 1.53	10.67 ± 2.52*	15.67 ± 2.08*#	7.33 ± 2.52*#^∧^	13.37 ± 1.93*^∧^
7	21.00 ± 4.00	9.67 ± 2.52*	14.67 ± 2.08*#	6.87 ± 1.89*#^∧^	12.33 ± 2.08*^∧^
14	20.33 ± 2.52	8.83 ± 2.57*	14.40 ± 1.91*#	6.40 ± 1.64*#^∧^	11.73 ± 1.70*^∧^
21	22.67 ± 1.53	8.63 ± 2.07*	14.00 ± 1.73*#	6.10 ± 2.23*#^∧^	11.43 ± 2.21*^∧^
28	21.67 ± 2.08	8.87 ± 1.67*	13.80 ± 1.65*#	6.30 ± 1.54*#^∧^	10.90 ± 2.15*^∧^

Compared with sham group, *p < 0.05; compared with model group, ^#^*p* < 0.05; compared with LXA4 group, ^∧^*p* < 0.05.

### LXA4 Decreased the Levels of Proinflammatory Factors and Increased Those of Anti-inflammatory Mediators

**FIGURE 2:**
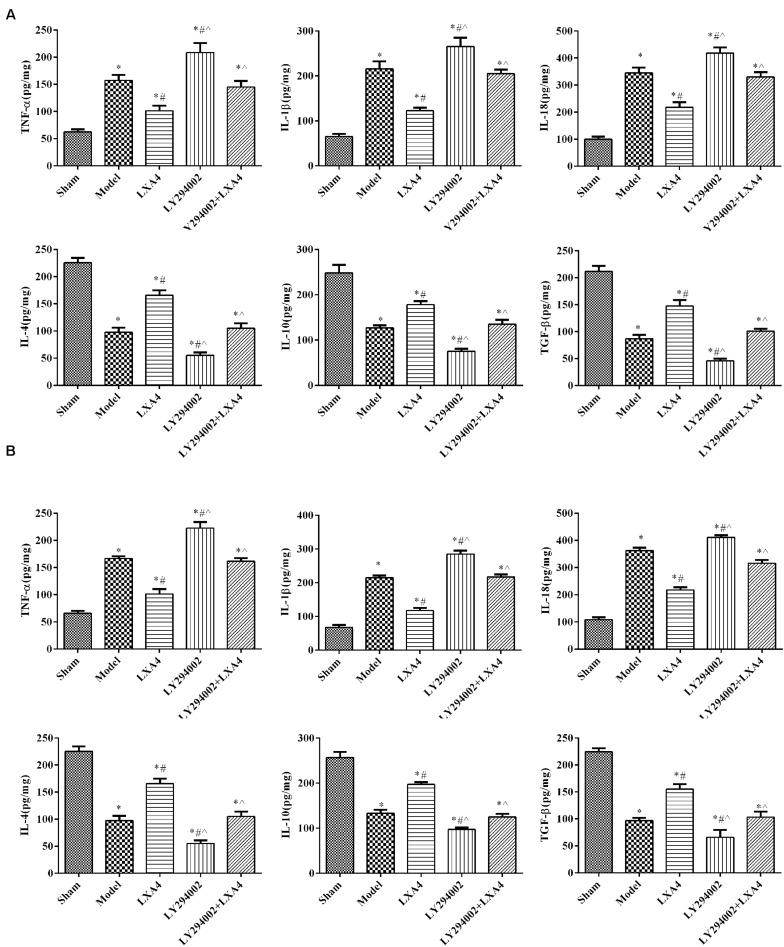
The expression of proinflammatory and anti-inflammatory mediators in the spinal dorsal horn **(A)** and dorsal root ganglion **(B)** by ELISA. *p < 0.05 compared with the sham group; #p < 0.05 compared with the model group; ^∧^*p* < 0.05 compared with the LXA4 group. Experiments were repeated three times for each group. Data are presented as means ± SD. One-way analysis of variance (ANOVA) was used for comparisons among groups followed by Dunnett’s *t*-test.

We investigated the levels of proinflammatory and anti-inflammatory mediators in the spinal dorsal horn (Figure 2A) and dorsal root ganglion (Figure 2B) by ELISA. In the spinal dorsal horn (Figure 2A), the expression levels of TNF-α, IL-1β, and IL-18 were significantly higher in the model group than in the sham group (*p* < 0.05). After LXA4 treatment, the levels of these proinflammatory factors were significantly reduced in the spinal dorsal horn of surgery rats (*p* < 0.05). We also found that injection of LY294002 led to an increase in the secretion of proinflammatory factors, while LXA4 injection weakened the effect of LY294002 (*p* < 0.05). Similar results were also recorded for the dorsal root ganglion (Figure 2B). For anti-inflammatory mediators in the spinal dorsal horn (Figure 2A), the expression of IL-4, IL-10, and TGF-β was significantly decreased in the surgery groups when compared with the sham group (*p* < 0.05). Among the surgery groups, the levels of the anti-inflammatory mediators were highest in the LXA4-treated group and lowest in the LY294002-injected group. Similarly, LAX4 treatment led to a significant increase in the contents of anti-inflammatory factors and weakened the effect of LY294002 in the dorsal root ganglion (Figure 2B, *p* < 0.05). Taken together, these results suggested that treatment with LXA4 could effectively regulate the levels of inflammatory factors in rats with non-compressive disc herniation.

### LXA4 Reduced the Expression of NLRP3 via the JNK1 Pathway in Rats With Non-compressive Disc Herniation

**FIGURE 3:**
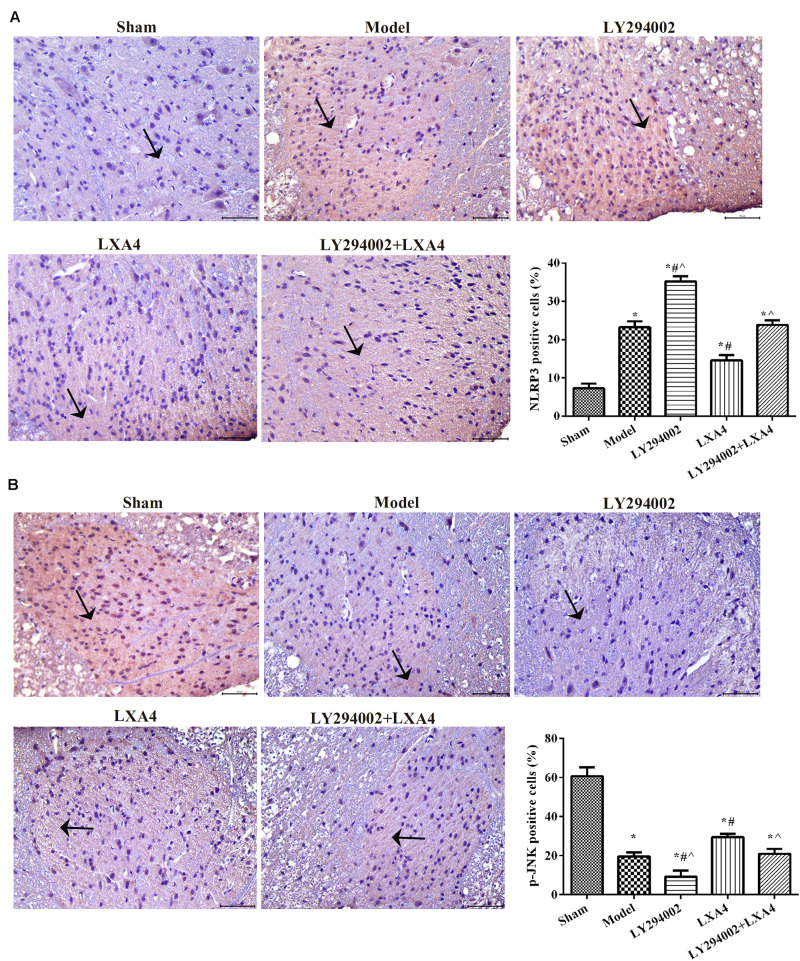
Immunohistochemical analysis of NLRP3 **(A)** and p-JNK1 **(B)** expression in the spinal dorsal horn. Magnification: × 400. Arrows indicate positive expression. *p < 0.05 compared with the sham group; ^#^*p* < 0.05 compared with the model group; ^∧^*p* < 0.05 compared with the LXA4 group. Experiments were repeated three times for each group. Data are presented as means ± SD. One-way analysis of variance (ANOVA) was used for comparisons among groups followed by Dunnett’s *t*-test.

**FIGURE 4:**
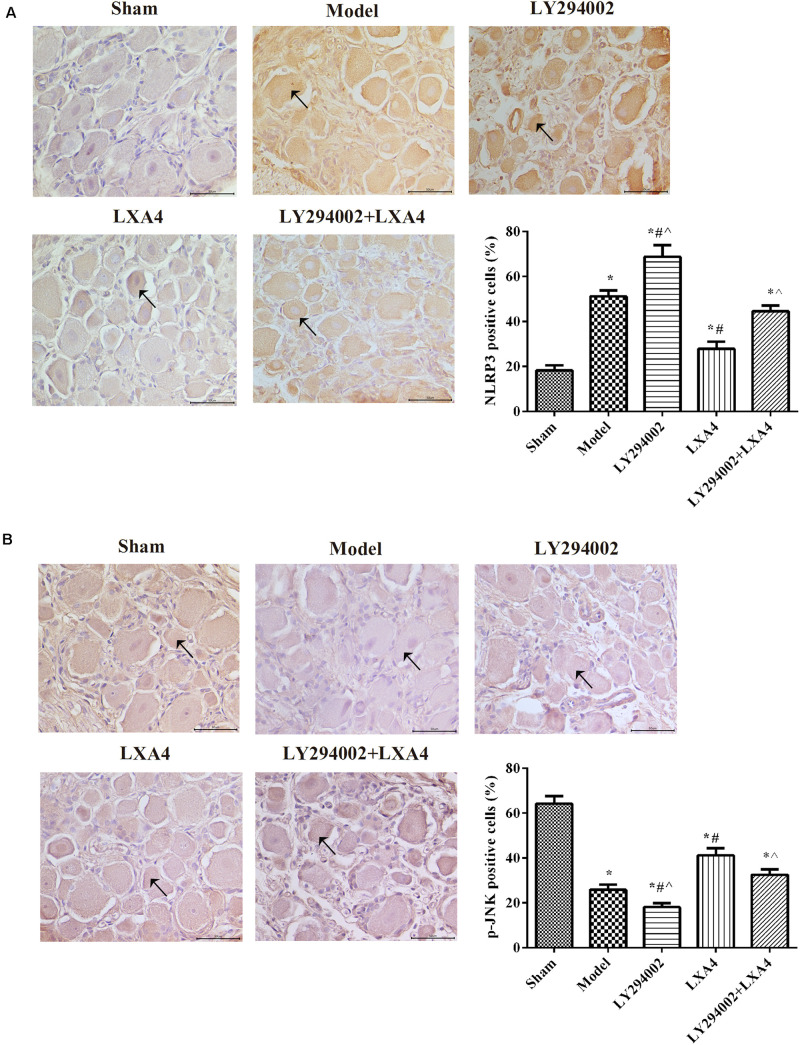
Immunohistochemical analysis of NLRP3 **(A)** and p-JNK1 **(B)** expression in the dorsal root ganglion. Magnification: × 400. Arrows indicate positive expression. *p < 0.05 compared with the sham group; ^#^*p* < 0.05 compared with the model group; ^∧^*p* < 0.05 compared with the LXA4 group. Experiments were repeated three times for each group. Data are presented as means ± SD. One-way analysis of variance (ANOVA) was used for comparisons among groups followed by Dunnett’s *t*-test.

We also analyzed the expression of NLRP3 and p-JNK1 in the spinal dorsal horn (Figure 3) and dorsal root ganglion (Figure 4) of rats by immunohistochemistry. As shown in Figures 3A, 4A, the expression of NLRP3 was greatly increased in the surgery groups compared with the sham group (*p* < 0.05). Injection of LY294002 further upregulated the expression of NLRP3 in the spinal dorsal horn of surgery rats. However, LXA4 administration decreased the expression level of NLRP3 and blocked the effect of LY294002 (*p* < 0.05). The expression of p-JNK1 was highest in the sham group and lowest in the LY294002 treatment group (*p* < 0.05). After surgery, the expression of p-JNK1 was significantly reduced, while LXA4 injection significantly increased its expression (*p* < 0.05). These results suggested that LXA4 suppresses NLRP3 activation and promotes p-JNK1 expression in rats with non-compressive disc herniation (Figures 3B, 4B).

### The Effect of LXA4 on the NLRP3 Inflammasome and Autophagy-Related Protein Expression *in vivo*

**FIGURE 5:**
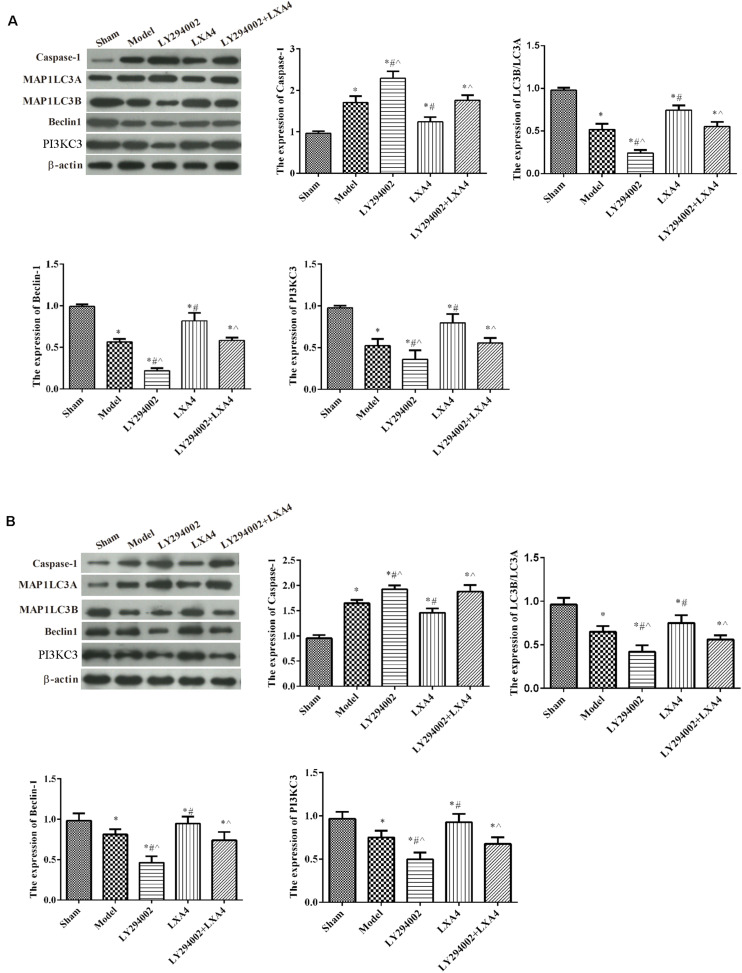
Western blot analysis of autophagy- and apoptosis-related protein expression in the spinal dorsal horn **(A)** and dorsal root ganglion **(B)**. *p < 0.05 compared with the sham group; ^#^*p* < 0.05 compared with the model group; ^∧^*p* < 0.05 compared with the LXA4 group. Experiments were repeated three times for each group. Data are presented as means ± SD. One-way analysis of variance (ANOVA) was used for comparisons among groups followed by Dunnett’s *t*-test.

The expression of the NLRP3 inflammasome and that of autophagy-related proteins in the spinal dorsal horn (Figure 5A) and dorsal root ganglion (Figure 5B) were measured by western blot. For caspase-1 expression, compared with the sham group, the expression of caspase-1 was significantly increased in the model group (*p* < 0.05), and adding LY294002 further increased caspase-1 expression. Injection of LXA4 significantly reduced the expression of caspase-1 and weakened the effect of LY294002 (*p* < 0.05). For autophagy-related proteins, compared with the sham group, the expression of MAP1LC3B/MAP1LC3A, beclin-1, and PI3KC3 was significantly decreased in the model group (*p* < 0.05). LY294002 administration further decreased the expression levels of these proteins. Meanwhile, treatment with LXA4 significantly increased the expression of autophagy-related proteins and diminished the effect of LY294002 (*p* < 0.05). These data suggested that LXA4 inhibited NLRP3 activation in spinal neuronal injury through the beclin-1/PI3KC3 pathway.

### The Effect of LXA4 on the Expression of Autophagy-Related Proteins in TNF-α-Induced Neuronal Cells *in vitro*

**FIGURE 6:**
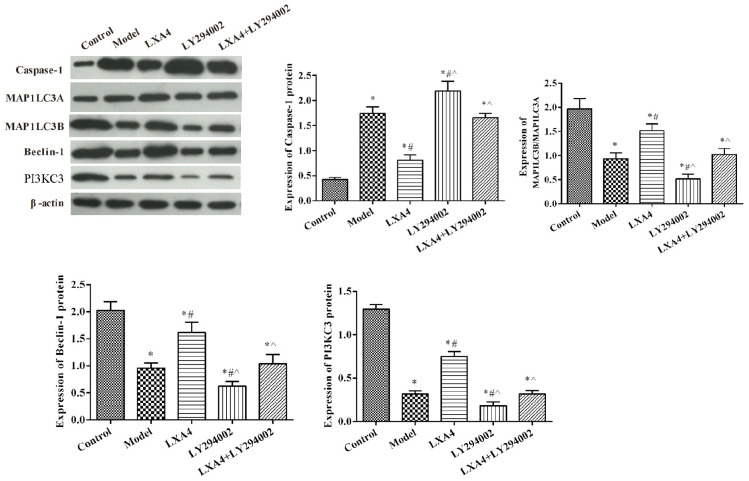
The expression of proinflammatory and anti-inflammatory mediators in spinal neuron cells by ELISA **(A)** and autophagy- and apoptosis-related protein expression in spinal neuron cells by western blot **(B)**. *p < 0.05 compared with the control group; ^#^*p* < 0.05 compared with the model group; ^∧^*p* < 0.05 compared with the LXA4 group. Experiments were repeated three times for each group. Data are presented as means ± SD. One-way analysis of variance (ANOVA) was used for comparisons among groups followed by Dunnett’s *t*-test.

The expression of autophagy-related proteins were also measured *in vitro* (Figure 6). Compared with the control group, the expression of MAP1LC3B/MAP1LC3A, Beclin-1, and PI3KC3 was significantly decreased in other groups (*p* < 0.05). The expression of caspase-1 was significantly increased after TNF-α stimulated, compared with control group (*p* < 0.05). LY294002 administration further decreased the expression levels of these proteins. Meanwhile, treatment with LXA4 significantly increased the expression of autophagy-related proteins and weakened the effect of LY294002 (*p* < 0.05).

### LXA4 Enhanced NSE and p-JNK1 Expression in TNF-α-Induced Neuronal Cells *in vitro*

**FIGURE 7:**
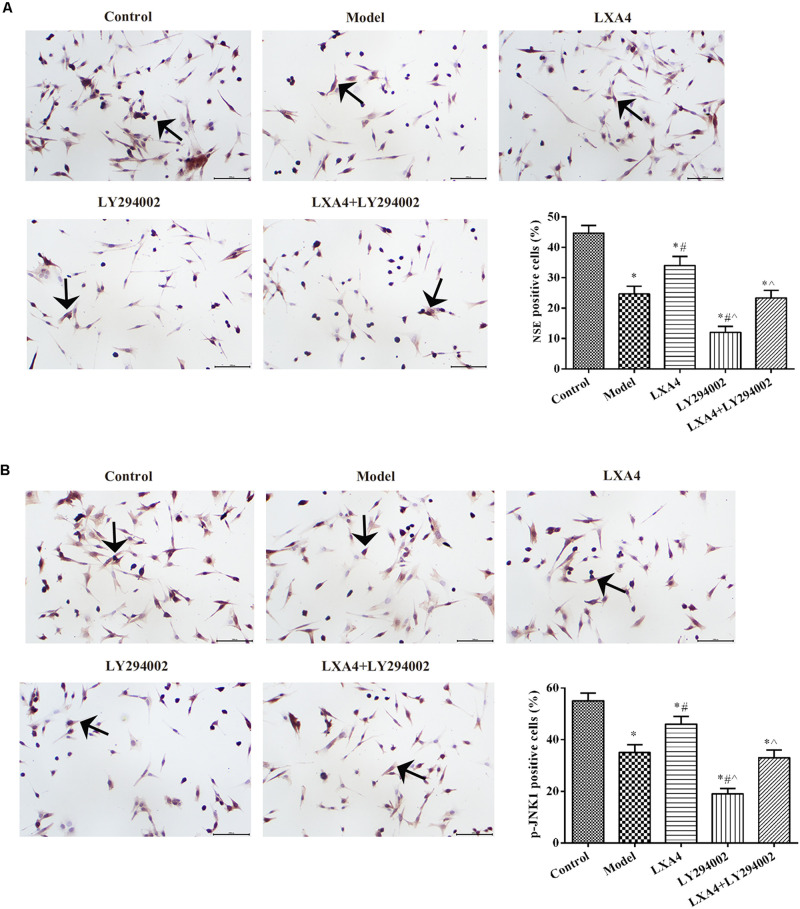
The expression of NSE **(A)** and p-JNK1 **(B)** in spinal neuron cells by immunohistochemistry. Magnification: × 400. Arrows indicate positive expression. *p < 0.05 compared with the control group; ^#^*p* < 0.05 compared with the model group; ^∧^*p* < 0.05 compared with the LXA4 group. Experiments were repeated three times for each group. Data are presented as means ± SD. One-way analysis of variance (ANOVA) was used for comparisons among groups followed by Dunnett’s *t*-test.

The expression of NSE and p-JNK1 in neuronal cells was measured by immunohistochemistry (Figure 7). NSE expression was notably decreased in TNF-α-induced spinal neurons when compared with control cells (*p* < 0.05) (Figure 7A). Following LXA4 treatment, NSE expression showed a significant increase (*p* < 0.05). When added alone, LY294002 elicited the opposite effect. Interestingly, however, in co-treated neuronal cells, LXA4 administration weakened the effect of LY294002. Additionally, the levels of p-JNK1 in spinal neuronal cells were highest in the control group and lowest in the LY294002 treatment group (*p* < 0.05) (Figure 7B). LXA4 administration increased the expression of p-JNK1 and suppressed the effect of LY294002 in TNF-induced cells. These data further indicated that LXA4 might improve spinal neuronal injuries through promoting p-JNK1 expression.

## Discussion

In this study, we found that intrathecal LXA4 injection could greatly improve the PWT in rats with non-compressive disc herniation. We also showed that LXA4 treatment effectively modulated NLRP3 inflammasome and autophagy-related protein levels via the JNK1 pathway both *in vivo* and *in vitro*.

Studies have shown that LXA4 inhibits the expression of proinflammatory factors, while increasing that of anti-inflammatory mediators (Luo et al., 2013). In addition, injection of exogenous LXA4 has been reported to help block TNF secretion, as well as significantly lower rat sensitivity to pain (Ariel et al., 2003). In this study, the data also showed that LXA4 can alleviate hyperalgesia in the spinal dorsal horn of rats. LXA4 treatment reduced the expression level of the inflammatory cytokines TNF, IL-1β, and IL-18. TNF is a proinflammatory cytokine known to increase the level of hyperalgesia, while IL-1β plays an important role in inducing neuropathic pain (Pociot et al., 1992). TGF-β is an important anti-inflammatory mediator (Shi and Massagué, 2003). In our study, the data were consistent with those of previous studies, and further confirmed the role and functions of LXA4 in regulating disc herniation through the downregulation of proinflammatory mediators and upregulation of anti-inflammatory factors.

In the current study, we also investigated the role of NLRP3 inflammasome activation in the dorsal horn of the spinal cord. Caspase-1 is the effector of the NLRP3 inflammasome. Formerly known as IL-1-converting enzyme, caspase-1 mediates IL-1β maturation and is critical for the regulation of the dorsal horn of the spinal cord (Doyle et al., 2019). In this study, we found that, compared with the model group, the expression of caspase-1 was decreased following LXA4 treatment, both *in vivo* and *in vitro*. LXA4 treatment was recently reported to reduce NLRP3 inflammasome-mediated IL-1β and IL-18 production in osteoclast-mediated diabetic osteoporosis (An et al., 2019). Abnormal activation of caspase-1 is associated with the aberrant activation of inflammasomes caused by ligands and IL-1β release. Here, we observed NLRP3 immune-reactivity in neurons of the spinal cord dorsal horn after the induction of non-compressive disc herniation in rats. Furthermore, the addition of the PI3K inhibitor supported that LXA4 regulates the NLRP3 inflammasome and JNK1 pathway, both *in vivo* and *in vitro*.

To further elucidate how LXA4 signaling affects radicular pain in rats, we assessed the changes in JNK phosphorylation status and expression levels of autophagy-related proteins. The expression of autophagy-related genes was shown to be markedly altered after LXA4 administration, and involved the conversion of cytosolic LC3 I to LC3 II and reduction of beclin-1 (Jia et al., 2015). In 2012, Manassero and colleagues reported that p-JNK1/JNK1 are significant regulators of tissue- and nerve injury-induced pain (Manassero et al., 2012). More recently, several studies have shown that autophagy is required for the precise regulation of the inflammatory response through suppressing the overstimulation of inflammatory responses, thereby avoiding damage to the body. A close relationship exists between autophagy and inflammasome activation (An et al., 2019; Doyle et al., 2019). In early autophagy, the PI3KC3 complex phosphorylates phosphatidylinositol to generate PI3P. In this complex, beclin-1, as a platform molecule, binds to PI3KC3, which can mediate the localization of autophagy-related proteins to phagocytic vesicles, thereby promoting the autophagic process (Fimia et al., 2007). Normally, Bcl-2 and beclin-1 interact and inhibit autophagy, and phosphorylation of Bcl-2 in autophagy has been proposed to be primarily mediated by JNK1 activation (Wei et al., 2008). Here, we found that intrathecal drug delivery of LXA4 promoted the expression of the autophagy-related proteins MAP1LC3B/MAP1LC3A, beclin-1, and PI3KC3. Our results further confirmed that LXA4 treatment increased the levels of p-JNK1/JNK1 and alleviated root pain in rats with non-compressive disc herniation. These results suggested that LXA4 may inhibit NLRP3 inflammasome activity *via* the JNK1/beclin1/PI3KC3 axis. Overall, our results support those of previous studies on the functions of LXA4 in non-compressive disc herniation.

Although the above results suggested that LXA4 clearly improved radicular pain in rats with non-compressive disc herniation via the JNK1/beclin1/PI3KC3 axis, this study has many limitations. The PI3KC3 may be the upstream of the JNK signaling pathway, and the relationship between JNK and PI3KC3 should be further identified. The autophagy-related protein levels are associated with the JNK1 pathway, and further mechanisms need to be researched.

In summary, delivery of exogenous LXA4 significantly alleviated radicular pain in rats with non-compressive disc herniation. LXA4 may exert its effects through reducing NLRP3 inflammasome activation, promoting the production of anti-inflammatory factors, and increasing the activity of JNK1, beclin-1, and PI3KC3.

## Data Availability Statement

The raw data supporting the conclusions of this manuscript will be made available by the authors, without undue reservation, to any qualified researcher.

## Ethics Statement

The animal experiments were carried following the NIH guidelines (NIH Pub. No. 85–23, revised 1996), and have been reviewed and approved by the Animal Protection and Use Committee of Shandong University.

## Author Contributions

JJ, YX, and CS designed the study and drafted the manuscript. YX, TS, and JM performed the experiments. YW, LQ, and KL performed the statistical analysis. All authors read and approved the final manuscript.

## Conflict of Interest

The authors declare that the research was conducted in the absence of any commercial or financial relationships that could be construed as a potential conflict of interest.
