# Corrigendum: Lipoxin A4 Inhibits NLRP3 Inflammasome Activation in Rats With Non-compressive Disc Herniation Through the JNK1/Beclin-1/PI3KC3 Pathway

**DOI:** 10.3389/fnins.2020.608184

**Published:** 2020-11-16

**Authors:** Jin Jin, Yonggang Xie, Cunxian Shi, Jiahai Ma, Yihao Wang, Leyan Qiao, Kezhong Li, Tao Sun

**Affiliations:** ^1^Department of Pain Management, Shandong Provincial Hospital, Cheeloo College of Medicine, Shandong University, Jinan, China; ^2^Department of Anesthesiology, The Affiliated Yantai Yuhuangding Hospital of Qingdao University, Yantai, China; ^3^Department of Anesthesiology, Qingdao Municipal Hospital, Qingdao, China

**Keywords:** lipoxin A4, non-compressive disc herniation, NLRP3, JNK1, beclin-1, PI3KC3

In the original article, there was a mistake in the legend for ^**^Figure 6^**^ as published. ^**^The results were error in Figure 6A**, so the legend of** Figure 6 was revised^**^. The correct legend appears below.

^**^Figure 6. The expression of autophagy- and apoptosis-related protein expression in spinal neuron cells by western blot. ^*^p < 0.05 compared with the control group; ^#^*p* < 0.05 compared with the model group; ^∧^*p* < 0.05 compared with the LXA4 group. Experiments were repeated three times for each group. Data are presented as means ± SD. One-way analysis of variance (ANOVA) was used for comparisons among groups followed by Dunnett's *t*-test. ^**^

In the original article, there was a mistake in ^**^Figure 6^**^ as published. ^**^The results of Figure 6A **were error, so the results of** Figure 6A **were deleted in** Figure 6 ^**^. The corrected ^*^Figure 6^**^ appears below.

**Figure 6:**
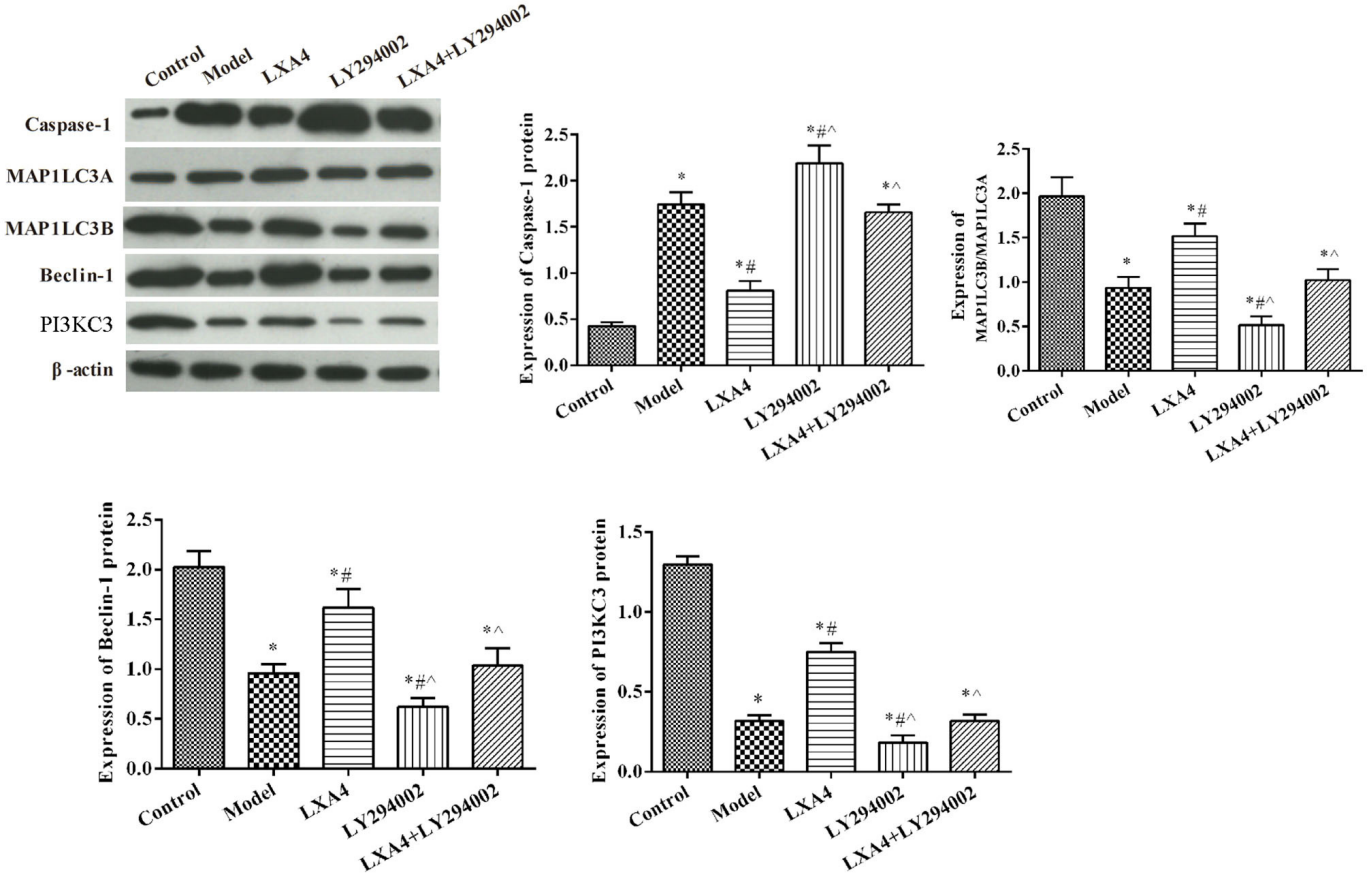
The expression of autophagy- and apoptosis-related protein expression in spinal neuron cells by western blot. ^*^p < 0.05 compared with the control group; ^#^*p* < 0.05 compared with the model group; ^∧^*p* < 0.05 compared with the LXA4 group. Experiments were repeated three times for each group. Data are presented as means ± SD. One-way analysis of variance (ANOVA) was used for comparisons among groups followed by Dunnett's *t*-test.

In the original article, there was an error. ^**^The levels of TNF (orb79138-480), IL-1β (orb79117), IL-18 (orb107403), IL-4 (orb303658), IL-10 (orb76364), and TGF-β1 (orb7087) (all from Biorbyt, Cambridge, United Kingdom) in the spinal dorsal horn, dorsal root ganglion, and spinal neurons were measured following the instructions of the respective ELISA kits.^**^

A correction has been made to ^**^Materials and Methods^**^, ^**^ELISA^**^, ^**^Page 3^**^:

^**^The levels of TNF-α (orb452907), IL-1β (orb453587), IL-18 (orb107403), IL-4 (orb303658), IL-10 (orb76364), and TGF-β1 (orb7087) (all from Biorbyt, Cambridge, United Kingdom) in the spinal dorsal horn and dorsal root ganglion were measured following the instructions of the respective ELISA kits. ^**^

In the original article, there was an error. ^**^The Effect of LXA4 on the Expression of the NLRP3 Inflammasome and Autophagy-Related Proteins in TNF-α-Induced Neuronal Cells in vitro^**^

A correction has been made to ^**^Results^**^, ^**^The Effect of LXA4 on the Expression of the NLRP3 Inflammasome and Autophagy-Related Proteins in TNF-α-Induced Neuronal Cells in vitro^**^, ^**^Page 9^**^:

^**^The Effect of LXA4 on the Expression of Autophagy-Related Proteins in TNF-α-Induced Neuronal Cells in vitro^**^

In the original article, there was an error. ^**^The levels of proinflammatory (TNF-α, IL-1β, and IL-18) and anti-inflammatory (IL-4, IL-10, TGF-β) mediators are shown in Figure 6A. Compared with control group, the levels of TNF-α, IL-1β, and IL-18 clearly increased in other groups (*p* < 0.05). Administration of LXA4 led to a marked reduction in the expression levels of TNF-α, IL-1β, and IL-18 compared with the model group, while the effect of LXA4 was weakened by LY294002 (*p* < 0.05). Compared with control group, the levels of TNF-α, IL-1β, and IL-18 clearly obviously decreased in other groups (*p* < 0.05). Meanwhile, the expression of IL-4, IL-10, and TGF-β was significantly increased in the LXA4 group compared with the model group (*p* < 0.05). Similar to the *in vivo* results, LAX4 treatment markedly upregulated the contents of anti-inflammatory factors and weakened the effect of LY294002. The expression of autophagy-related proteins were also measured *in vitro* (Figure 6B). Compared with the control group, the expression of MAP1LC3B/MAP1LC3A, Beclin-1, and PI3KC3 was significantly decreased in other groups *(p* < 0.05). The expression of caspase-1 was significantly increased after TNF-α stimulated, compared with control group (*p* < 0.05). LY294002 administration further decreased the expression levels of these proteins. Meanwhile, treatment with LXA4 significantly increased the expression of autophagy-related proteins and weakened the effect of LY294002 (*p* < 0.05).^**^

A correction has been made to ^**^Results^**^, ^**^The Effect of LXA4 on the Expression of Autophagy-Related Proteins in TNF-α-Induced Neuronal Cells in vitro^**^, ^**^Page 10^**^:

^**^The expression of autophagy-related proteins were also measured *in vitro* (Figure 6). Compared with the control group, the expression of MAP1LC3B/MAP1LC3A, Beclin-1, and PI3KC3 was significantly decreased in other groups (*p* < 0.05). The expression of caspase-1 was significantly increased after TNF-α stimulated, compared with control group (*p* < 0.05). LY294002 administration further decreased the expression levels of these proteins. Meanwhile, treatment with LXA4 significantly increased the expression of autophagy-related proteins and weakened the effect of LY294002 (*p* < 0.05). ^**^

The authors apologize for this error and state that this does not change the scientific conclusions of the article in any way. The original article has been updated.

